# Supplementary material for: Carboxyl-Terminal Decoy Epitopes in the Capsid Protein of Porcine Circovirus Type 2 Are Immunogenicity-Enhancers That Elicit Predominantly Specific Antibodies in Non-Vaccinated Pigs
Source: Viruses. 2022 Oct 27;14(11):2373. doi: 10.3390/v14112373 (PMC9693397; doi:10.3390/v14112373)
Supplement: Supplementary file 1 [file viruses-14-02373-s001.zip › viruses-1946560-supplementary.pdf]

Supplementary Materials

The Decoy Epitopes Elicit Swine Antibodies by Connecting to the Carboxyl-terminal Epitope of the Capsid Protein of Porcine Circovirus Type 2

Ling-Chu Hung [1, 2, 3, †]

[1] Animal Drugs Inspection Branch, Animal Health Research Institute, Council of Agriculture, Executive Yuan, Miaoli County 35054, Taiwan; lchung@mail.nvri.gov.tw; Tel.: +88-637584811
[2] Animal Health Research Institute, Council of Agriculture, Executive Yuan, New Taipei City 25158, Taiwan.
[3] Livestock Research Institute, Council of Agriculture, Executive Yuan, Tainan 71246, Taiwan
† Current address: Animal Drugs Inspection Branch, Animal Health Research Institute, Council of Agriculture, Executive Yuan, Miaoli County 35054, Taiwan

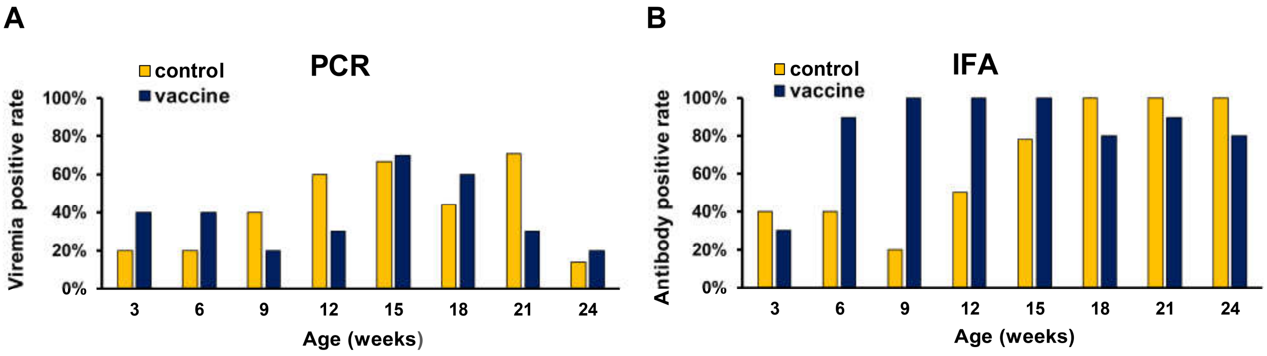

Figure S1. Both vaccinated (n=10) and control (n=10) swine sera had been examined with the immunofluorescence assay and polymerase chain reaction. The previous study herd had PCV2 infection without getting the PCV2 vaccine in the conventional pig farm with the farrow-to-finish operation [1]. Two piglets of the same sex and similar body weight (close to the mean body weight of the entire newborn litter) were selected from each litter. This study involved 20 newborn piglets (10 male & 10 female) of TLRI Black Pig No.1 (TBP), delivered from 10 sows between January and March 2011. One piglet from each litter was injected intramuscularly with the VLP-B vaccine (Ingelvac CircoFLEX®, 1.0 mL, one dose at 3 weeks of age). The other piglet from each litter was injected intramuscularly with 2.0 mL of saline at 3 weeks of age and served as a control. Blood samples from each pig were collected at 3, 6, 9, 12, 15, 18, 21, and 24 weeks of age. (A) The PCV2-DNA (viremia) positive rate in pig groups [1]. (B) The anti-PCV2 IgG (IFA) positive rate of PCV2 in pig groups [1].

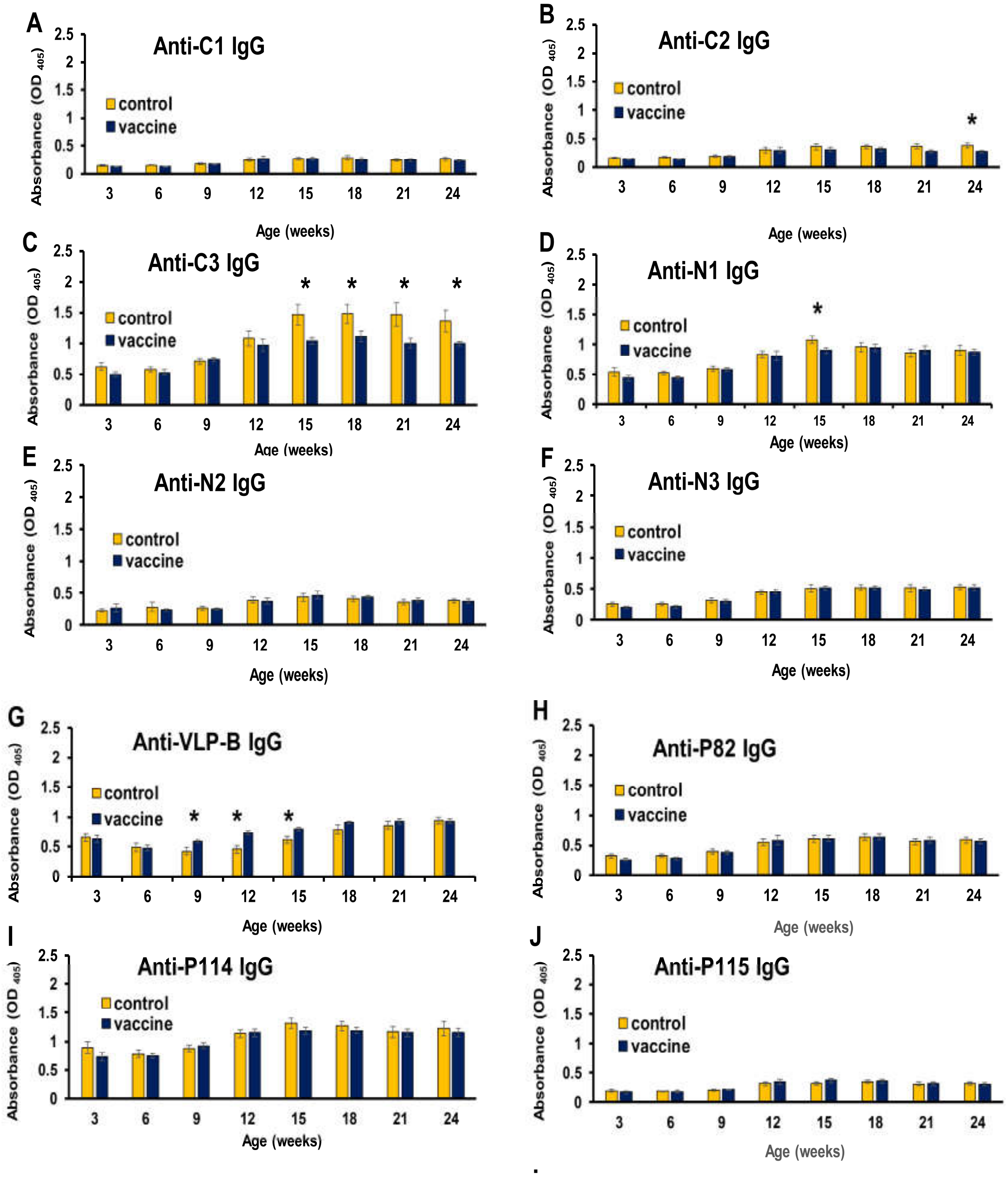

Figure S2. Comparison of anti-PCV2 peptide-specific IgGs between two groups of pigs at different ages. Among vaccinated (n=10) and control (n=10) animals, the anti-PCV2 peptide-specific IgGs were detected in the serum samples from pigs at different ages (3, 6, 9, 12, 15, 18, 21, and 24 weeks of age). (A–J) The viral peptides (the N-terminus of the capsid peptide (C1), the middle region of the capsid peptide (C2), the C-terminus of the capsid peptide (C3), the ORF3 peptide (N1), the ORF6 peptide (N2), the ORF9 peptide (N3), the binding residues (P82) of mAb 4F6, the ORF10 peptide (P114), and ORF11 peptide (P115) and the immunogen (VLP-B)) were used as coating antigens in iELISA test. The data were analyzed by using ANOVA and Tukey's Studentized Range multiple comparisons test using the SAS Enterprise Guide 7.1® software. The data represent the mean ± standard error. Significant p values are indicated as * $p < 0.05$.

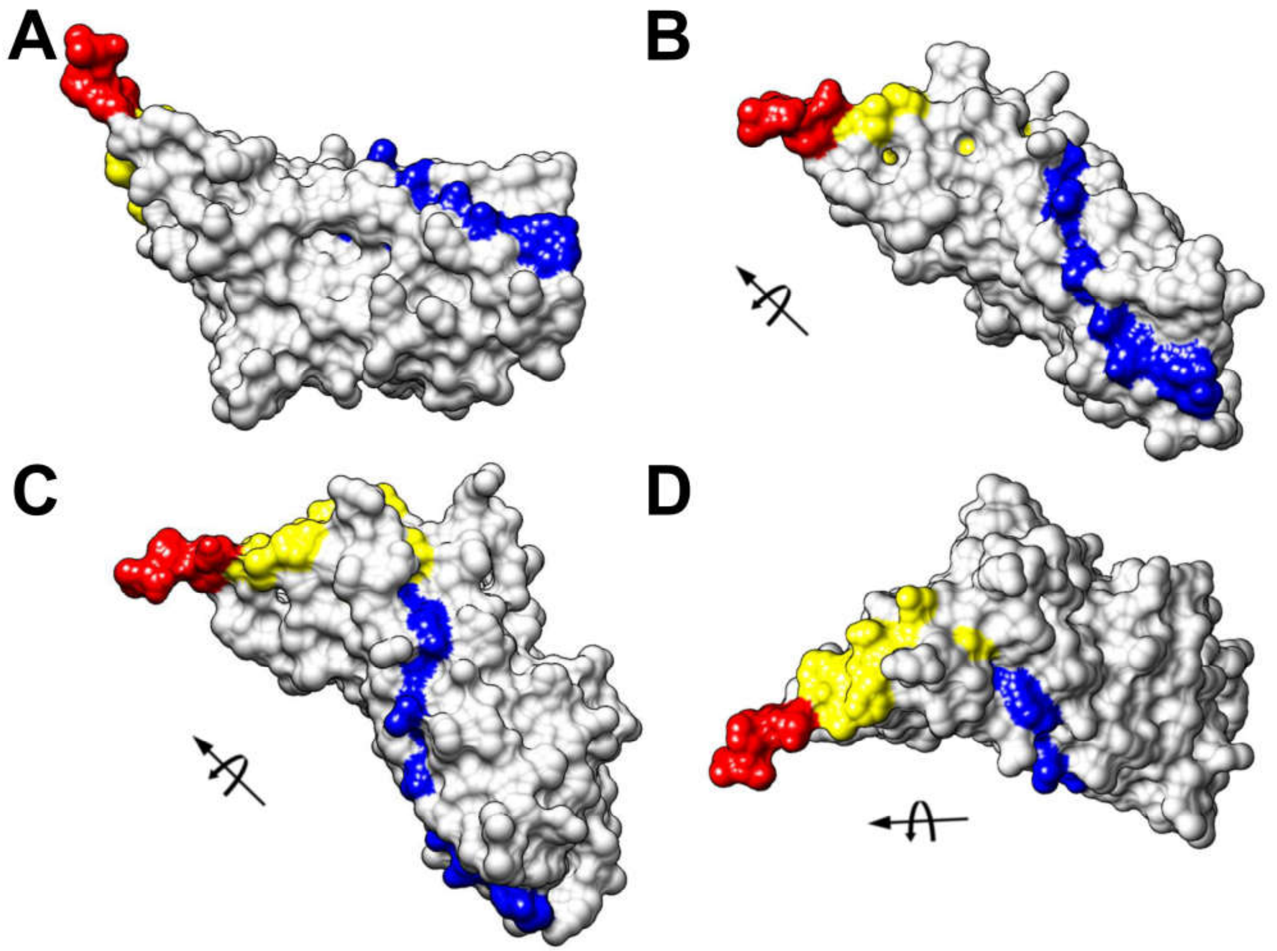

Figure S3. Location of amino acid residues of designed peptide (P64) and critical binding residues on the single capsid protein of PCV2a. The residues 228–231 were highlighted in red, the residues 220–227 were labeled in yellow, and the other residues 205–219 were labeled in blue. These color residues represented the peptide P64. (A–D) 3-D model of the single capsid protein subunit rendered as a solid surface looking at four different views. The structural model of the capsid protein of PCV2a coordinates was retrieved from the Protein Data Bank (PDB) entries for the capsid protein of PCV2a (PDB code: 3JCI) [2], and images were generated using UCSF Chimera version 1.14 from the Resource for Biocomputing, Visualization, and Informatics at the University of California, San Francisco, USA [3].

References

1. Hung, L.C.; Huang, Y.H.; Lai, Y.S.; Lee, W.C.; Wang, C.; Lin, Y.L.; Cheng, I.C. Humoral Immunity of TLRI Black Pig and the Effect of PCV2 Subunit Vaccination into It [畜試黑豬之體液免疫及接種環狀病毒次單位疫苗之研究]. *J Chin Soc Anim Sci* **2016**, *45*, 241–257.

2. Z, L.; F, G.; F, W.; Tc, L.; W, J. 2.9 Å Resolution Cryo-EM 3D Reconstruction of Close-Packed Virus Particles. *Structure* **2016**, *24*, 319–328, doi:10.1016/j.str.2015.12.006.
3. Pettersen, E.F.; Goddard, T.D.; Huang, C.C.; Couch, G.S.; Greenblatt, D.M.; Meng, E.C.; Ferrin, T.E. UCSF Chimera--a Visualization System for Exploratory Research and Analysis. *J Comput Chem* **2004**, *25*, 1605–1612, doi:10.1002/jcc.20084.
